# Kinetics of Anti-SARS-CoV-2 Antibody Responses 3 Months Post Complete Vaccination with BNT162b2; A Prospective Study in 283 Health Workers

**DOI:** 10.3390/cells10081942

**Published:** 2021-07-30

**Authors:** Evangelos Terpos, Ioannis P. Trougakos, Vangelis Karalis, Ioannis Ntanasis-Stathopoulos, Sentiljana Gumeni, Filia Apostolakou, Aimilia D. Sklirou, Maria Gavriatopoulou, Stamatia Skourti, Efstathios Kastritis, Eleni Korompoki, Ioannis Papassotiriou, Meletios A. Dimopoulos

**Affiliations:** 1 Department of Clinical Therapeutics, School of Medicine, National and Kapodistrian University of Athens, 11528 Athens, Greece; johnntanasis@med.uoa.gr (I.N.-S.); mgavria@med.uoa.gr (M.G.); tinaskou1995@hotmail.com (S.S.); ekastritis@gmail.com (E.K.); e.korompoki@imperial.ac.uk (E.K.); mdimop@med.uoa.gr (M.A.D.); 2 Department of Cell Biology and Biophysics, Faculty of Biology, National and Kapodistrian University of Athens, 11528 Athens, Greece; itrougakos@biol.uoa.gr (I.P.T.); sgumeni@biol.uoa.gr (S.G.); asklirou@biol.uoa.gr (A.D.S.); 3 Section of Pharmaceutical Technology, Department of Pharmacy, School of Health Sciences, National and Kapodistrian University of Athens, 11528 Athens, Greece; vkaralis@pharm.uoa.gr; 4 Department of Clinical Biochemistry, “Aghia Sophia” Children’s Hospital, 11527 Athens, Greece; fapostolakou@yahoo.gr (F.A.); ipapassotiriou@gmail.com (I.P.)

**Keywords:** COVID-19, SARS-CoV-2, neutralizing, antibodies, immunity, BNT162b2

## Abstract

The aim of this study was to investigate the kinetics of neutralizing antibodies (NAbs) and anti-SARS-CoV-2 anti-S-RBD IgGs up to three months after the second vaccination dose with the BNT162b2 mRNA vaccine. NAbs and anti-S-RBD levels were measured on days 1 (before the first vaccine shot), 8, 22 (before the second shot), 36, 50, and three months after the second vaccination (D111) (NCT04743388). 283 health workers were included in this study. NAbs showed a rapid increase from D8 to D36 at a constant rate of about 3% per day and reached a median (SD) of 97.2% (4.7) at D36. From D36 to D50, a slight decrease in NAbs values was detected and it became more prominent between D50 and D111 when the rate of decline was determined at −0.11 per day. The median (SD) NAbs value at D111 was 92.7% (11.8). A similar pattern was also observed for anti-S-RBD antibodies. Anti-S-RBDs showed a steeper increase during D22–D36 and a lower decline rate during D36–D111. Prior COVID-19 infection and younger age were associated with superior antibody responses over time. In conclusion, we found a persistent but declining anti-SARS-CoV-2 humoral immunity at 3 months following full vaccination with BNT162b2 in healthy individuals.

## 1. Introduction

The novel coronavirus severe acute respiratory syndrome coronavirus 2 (SARS-CoV-2) has led to a worldwide pandemic and has become a major global health concern. The coronavirus genome encodes four different main structural proteins designated as spike (S), envelope, membrane, and nucleocapsid. The virus penetrates through the viral S protein to the angiotensin-converting enzyme 2 (ACE2) receptors that are mainly presented on oral mucosa epithelial cells and lung alveolar type II cells, as well as in other human tissues [1,2]. COVID-19 is a systemic disease with both short- and long-term manifestations [3,4]. Most of the patients will present with mild or moderate symptoms, although up to 5–10% will present with severe or life-threatening disease course. The development of effective and safe vaccines, as well as novel therapeutics, has become a global priority [5,6,7]. 

The BNT162b2 vaccine offers high protection against COVID-19 [8,9,10,11]. Healthy individuals show high levels of the anti-SARS-CoV-2 Spike-receptor binding domain (anti-S-RBD) IgG antibodies and neutralizing antibodies (NAbs), as well as a persistent germinal center B-cell response following vaccination [10,12,13,14]. Importantly, NAbs levels correlate with clinically relevant immune protection from COVID-19 [15]. However, a slight decline in antibody titers has become evident even at one month following the second BNT162b2 shot [13], whereas an increased time since the second vaccine dose has been associated with decreased NAb activity against SARS-CoV-2 variants [16]. 

The aim of this study was to investigate the kinetics of NAbs and anti-S-RBD IgGs after vaccination of health workers with the BNT162b2 mRNA vaccine over a period of up to three months after the second shot. The possible influence of comorbidities, characteristics of the subjects, co-medication, and adverse events were also investigated. 

## 2. Materials and Methods

### 2.1. Clinical Procedures

All participants have been enrolled in a large prospective study (NCT04743388) evaluating the kinetics of anti-SARS-CoV-2 antibodies after COVID-19 vaccination. Here, we present data only in health workers of the Alexandra General hospital in Athens, Greece. According to the National Immunization Program, healthcare workers have been prioritized for vaccination since January 2021 and, therefore, mature data for the antibody responses at 3 months following vaccination have been collected. The study was in accordance with the Declaration of Helsinki and International Conference for harmonization for good clinical practice and was approved by the ethics committee of Alexandra Hospital. All subjects gave informed consent before participating in the study. 

The main inclusion criteria for participation in this study were: eligibility for vaccination according to the national program for COVID-19, age above 18 years, and the ability to sign the informed consent form. Major exclusion criteria included the presence of active malignant disease, immunosuppressive therapy, and end-stage renal disease. According to National Immunization Program, access to the BNT162b2 mRNA vaccine was available to anyone 18 years of age or older.

Subject data were kept confidential in accordance with the rules of the General Data Protection Regulation. All names were kept confidential. Immediately after collection, names were deleted and randomly replaced with a unique number so that the patient could not be identified.

### 2.2. Measurement of Antibodies

The schedules for blood collection were day 1 (D1) before the first vaccination, day 8, day 22 (the day of the second vaccination and just before receiving the injection), day 36 (namely, two weeks after the second vaccination), day 50 (one month later), and day 111 (i.e., three months after completion of the second vaccination). After vein puncture, serum was separated within 4 h of blood collection and stored at −80 °C until the day of measurement. Stored samples from different time points of the same donor were measured in parallel assays.

Anti-S-RBD IgG antibodies, which indicate response to either prior infection or vaccine, were measured using an FDA-approved method, namely, the Elecsys Anti-SARS-CoV-2 S assay (Roche Diagnostics GmbH, Mannheim, Germany). Neutralizing antibodies (NAbs) for SARS-CoV-2 were measured using an FDA-approved method. The cPass™ SARS-CoV-2 NAbs Detection Kit (GenScript, Piscataway, NJ, USA), which provides indirect detection of potential SARS-CoV-2 NAbs in blood was used. According to this method, antibody-mediated inhibition of SARS-CoV-2 RBD binding to the human host receptor angiotensin-converting enzyme type 2 is tested.

### 2.3. Data Organization

The anti-S-RBD IgG titters and the (%) inhibition of SARS-CoV-2 binding to the human host receptor angiotensin-converting enzyme-2 after vaccination with the BNT162b2 mRNA vaccine were measured. Based on these values, a newly introduced term called “Rate of Change” (RC) was calculated. RC refers to the average change in NAbs (or anti-S-RBD) values between two consecutive time points (days). Since the time scale in this analysis was “day”, the units of RC (%) are inhibition titers (or anti-S-RBD units/mL) of SARS-CoV-2 binding per day. In other words, RC refers to the rate of production or degradation of antibodies in the body. Positive values refer to production, negative values to elimination, while values close to zero indicate no change. The two time points referred to by RC are shown as subscripted numbers next to this symbol; for example, the expression RC36–50 is the rate of change from day 36 to 50. The variable RC was introduced to better reflect the change in antibody scores. In this study, RC was calculated for all time intervals between two consecutive days, and a visual representation of each RC value is provided in the subsequent analysis. Since the measurements of NAbs were taken on six occasions, there were five-time intervals and the points refer to the RC value of each subject who participated in the study.

Demographic data, comorbidities, and any medications taken were collected from patients after personal communication with them. Body mass index (BMI) was calculated using each individual’s weight and height data. Based on BMI, the subjects were divided into groups: Underweight with a BMI of less than 18.4, Normal weight with a BMI of 18.5 to 24.9, Overweight: BMI 25 to 29.9, and Obese with a BMI of 30 or more. 

The medical history of the subjects included various diseases, which were classified into the following groups: Hypercholesterolemia (e.g., dyslipidemia), cardiovascular disease (e.g., hypertension, coronary artery disease), diabetes, autoimmune diseases (e.g., psoriasis, atopic dermatitis, irritable bowel syndrome), allergies (seasonal, bronchial asthma, chronic obstructive pulmonary disease, previous reactions to medications), thyroid problems (e. e.g., Hashimoto’s, hypothyroidism), and others (e.g., depression, migraine, sleep apnea, gastroesophageal reflux disease). Similarly, all medications received were classified into the classes: Hypercholesterolemia medications (e.g., statins), cardiovascular disease medications (beta-blockers, ACE inhibitors I and II), insulin and oral antidiabetics, T4, antihistamines and/or inhaled corticosteroids, immunomodulators, centrally acting drugs (e.g., benzodiazepines, antidepressants), and others (such as acetylsalicylic acid, proton pump inhibitors, nonsteroidal anti-inflammatory drugs, vitamins, etc.).

Post-vaccination adverse events were classified into the following groups: local effects (e.g., pain or swelling at the vaccination site, limitation of hand movement), fatigue, arthralgias/myalgias/chills/fever, headache, dizziness/sleepiness, allergies (such as itching, runny nose, flushing), anaphylaxis, and others that did not fall into any of the above categories.

### 2.4. Statistical Analysis

Statistical analysis began with descriptive criteria such as mean, median, quartiles, and estimation of dispersion metrics. Before statistical comparisons were made between two or more groups, a normality test took place. The Kolmogorov–Smirnov and Shapiro–Wilk tests and QQ plots were used to assess the normality of the data distribution. According to these tests, if the nominal normality hypothesis is rejected, the data is considered not to follow the normal distribution. In all cases of this analysis, the data was found to deviate from normality (see Tables S1–S4 in the Supplementary material). Therefore, non-parametric methods were used for the subsequent statistical analysis. For two independent group comparisons, such as assessing the gender effect or the influence of age groups (<50 and ≥50 years), the Mann–Whitney U test was used. For pairwise group comparisons, such as neutralizing antibody levels between two occasions, the Wilcoxon signed-rank test was used. The non-parametric Friedman test was used to detect differences between individuals (e.g., in their NAbs or anti-SRBD titers) across multiple time points, i.e., days 36, 50, and 111. Chi-square analysis was used for comparisons of nominal characteristics, such as the occurrence of adverse events after the first and second vaccinations. 

In addition, principal component analysis (PCA) was used. PCA is a dimensionality reduction method used to reduce the dimensionality of data sets (i.e., the number of variables). The new coordinates of the data sets in the dimensionality reduced space denote the “scores”, while the “loadings” correspond to the correlations between the original variables and the new dimensions (components). Loading values close to +1 or −1 mean that the variable strongly influences the component, while estimates close to zero indicate that the variable has a weak or no influence on the component. The input data were centered, and singular value decomposition was applied. Biplots were created to show the scores and loadings in a single plot. 

In all cases in this study, the significance level was set at 5% and a result was considered significant if the estimated *p*-value (p) was less than the significance level. Statistical analysis was performed in Python v.3.9.2. The “pandas” and “numpy” libraries were used for data analysis. Statistical plots were created using the library “matplotlib”, while statistics and PCA analysis were also implemented using the libraries “seaborn”, “sklearn” and “bioinfokit”.

## 3. Results

### 3.1. Baseline Characteristics

This study included 283 health workers (median age 48 years), who received two doses of the BNT162b2 mRNA vaccine. The demographic data of the participants are shown in Table 1. 

### 3.2. Kinetics of NAbs

**Figure 1:**
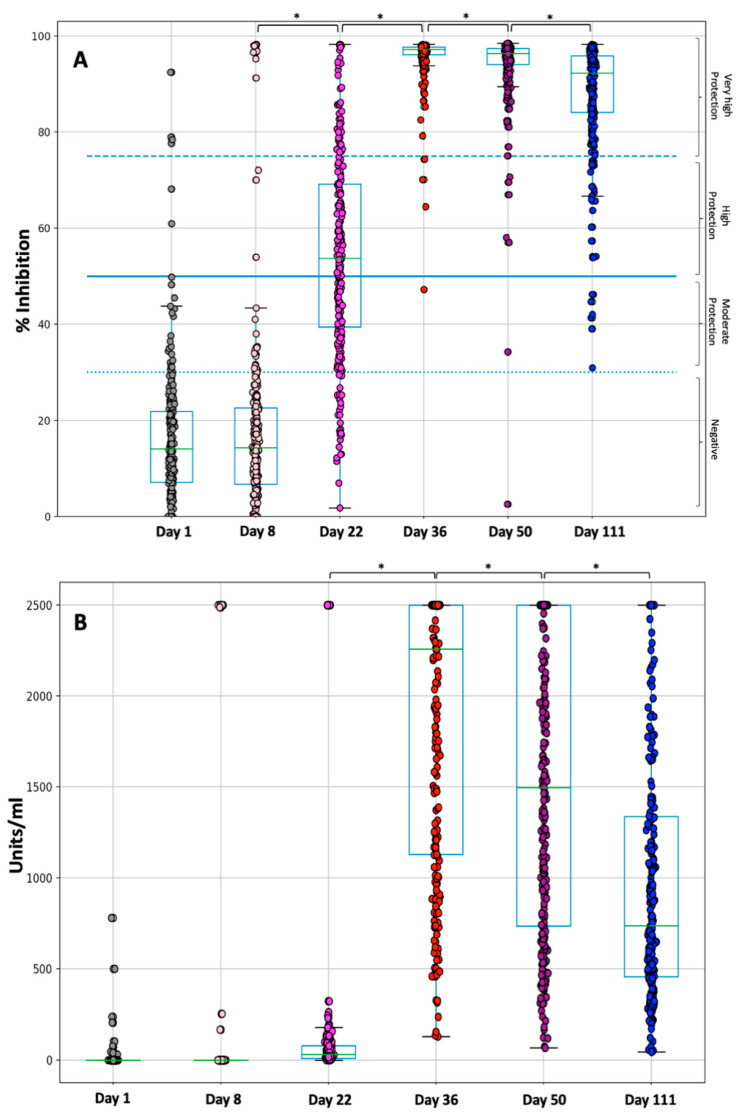
Inhibition (%) of SARS-CoV-2 binding to the human host receptor angiotensin-converting enzyme-2 (**A**) and anti-SRBD levels (**B**) after vaccination with the BNT162b2 mRNA vaccine. Antibodies were measured on D1 (vaccination date), D8, D22, D36, D50, and D111 (3 months after the second shot). Asterisks (*) indicate statistically significant differences (*p*-value < 0.05) between the compared groups. The boxplot borders refer to the quartiles of the distribution, while the overlaid dots represent the individual values of NAb inhibition. Dashed lines refer to the borderline levels of inhibition i.e., 30%, 50%, and 75%.

Figure 1A shows the percent inhibition of NAbs on D1, 8, 22, 36, 50, and 111 following the first dose of BNT162b2. On D1, immediately before vaccination, the median neutralizing inhibition was 14.2%, while 29 individuals (10.2%) had inhibition levels above the threshold of 30%. There were also 5 individuals (1.8%) with values corresponding to very high protection (NAbs titer above 75%). At D8, i.e., seven days after vaccination, NAb values remained almost the same (median 14.3%), while three weeks later (D22) and before the second vaccination, titers were significantly increased (median 53.8%). At D8, the mean titers were 16.0% and 39.7% for the individuals who had never had PCR+ for SARS-CoV-2 and for those who had PCR+ and COVID-19 in the past, respectively. This shows the strong influence of the previous COVID-19 on the production of NAbs. This effect remains eminent at D22, where the mean inhibitory values are still much higher (65.6% vs. 54.1%) for the previously PCR+ subjects. As expected, two weeks later (i.e., D36), NAbs titers increased in the vast majority of individuals (median 97.2%); the latter is reflected in the low dispersion [standard deviation (SD) 4.7] of scores observed at D36. After D36, however, NAbs levels began to decline in some participants. One month after completion of vaccination with the second shot (i.e., on D50), although half of the subjects (median 96.3%) still had the same high inhibition scores, there were many subjects with lower titers, as reflected in the wide dispersion of scores (SD almost doubled to 8.5). 

At three months after the second vaccination (i.e., on D111), the decline in NAb titers was even more prominent with a median inhibition of 92.7% (SD 11.8). At this point, 7 subjects (2.5%) had inhibition levels of less than 50%, which is considered as the cut-off of clinically significant protection against SARS-CoV-2. Of note, none of the subjects had values below 30% (positive threshold of the method), indicating that all subjects had at least mild/moderate protection against SARS-CoV-2. Overall, it appears that maximum protection occurs two weeks after the second vaccination, with a steady, slow decline in NAbs levels thereafter. Paired grouped comparisons using the Wilcoxon signed ranks test showed the statistically significant differences in inhibition levels between pairs: D36 vs. D50, D36 vs. D111, and D50 vs. D111 (for all three comparisons *p* < 0.001). The same result of a significant difference was obtained when the three groups (D36, D50, D111) were compared simultaneously using the Friedman test (*p* < 0.001). In Table S5 of the supplementary material, the abovementioned statistical results are quoted. 

### 3.3. Kinetics of Anti-S-RBDs

A similar pattern was also observed for anti-S-RBD antibodies (Figure 1B). At D1, 254 (90%) individuals had anti-S-RBD levels less than 20 units/mL, i.e., they were considered “negative”. Five subjects (2%) were considered to have moderate protection (20 to 90 units/mL), while only 5 (2%) exhibited high protection (higher than 90 units/mL). The same profile was still obvious up to D22. However, at D36 a rapid increase in the anti-S-RBD levels was observed and the median anti-S-RBD was 2304 units/mL. At this timepoint, all individuals had anti-S-RBD titers above 90 units/mL. After D36, a steep decline in anti-S-RBD titters is observed where the median antibody levels were 1504 and 761 units/mL at D50 and D111, respectively. It is noteworthy that three months after vaccination, only 4 individuals had anti-S-RBDs less than the critical value of 90 units/mL; namely, 98.6% of them were still considered as highly protected. The statistical assessment revealed significant differences in anti-S-RBD from D22 to D111 (Friedman’s test *p*-value < 0.001). Also, all consecutive pairs comparison, using Wilcoxon’s test, led to *p*-values < 0.001 (Figure 1B). In Table S6 of the supplementary material, the above-mentioned statistical results are quoted.

### 3.4. NAbs and Anti-S-RBDs: Rate of Change

**Figure 2:**
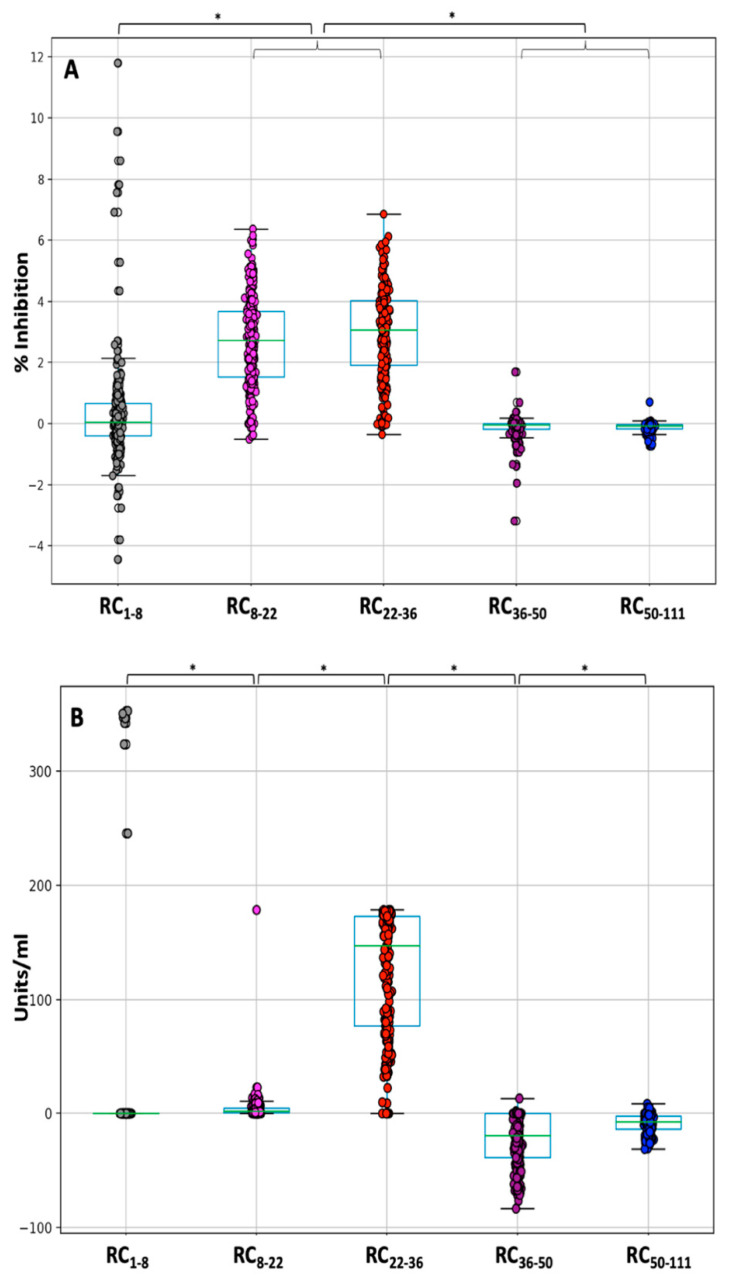
Rate of change in the (%) inhibition of SARS-CoV-2 binding to the human host receptor angiotensin-converting enzyme-2 (**A**) and anti-SRBD levels (**B**) after vaccination with the BNT162b2 mRNA vaccine. Rate of change (RC) values express the average change of antibody titers between two occasions and were estimated for two subsequent time points: RC_1–8_, RC_8–22_, RC_22–36_, RC_36–50_, RC_50–111_. Positive values indicate an increase in inhibition, negative values imply a decrease, and values close to zero show no change in the (%) inhibition of SARS-CoV-2 binding. Asterisks (*) indicate statistically significant differences (*p*-value < 0.05) between the compared groups.

To further examine the kinetics of antibody response, the “rate of change (RC)” was estimated. Regarding the NAb levels, Figure 2A shows that in the period between D1 and D8 the RC_1–8_ values range from −4.5 to 11.8. Negative RC values correspond to subjects with NAbs on D1 since they had COVID-19 in the past (PCR+). Regardless of these subjects, a small and variable increase in NAb production was observed with an average value of 0.29 (median 0.06, SD 1.7). However, the increase became evident from D8 to D36. In both intervals (8–22 and 22–36), RC gets almost the same value (mean values 2.7 and 2.9, respectively), highlighting the uniform and rapid increase in NAb inhibition. Thus, after one week of the first vaccination until two weeks after the second vaccination, the production of NAbs was high and on average the inhibition increases at a constant rate of about 3% per day. It was found that the high rate of production of NAbs throughout the period of D8-D22-D36 was statistically significantly different from the initial period of one week from the first day of vaccination (Friedman test *p*-value < 0.001). However, from D36 to D50, there is a small decrease in NAbs values. In fact, the average decrease was −0.13 per day, with half of the subjects having a decrease of −0.05 per day. The same pattern was also observed between D50 and D111, i.e., between one month and three months after completion of vaccination. During this period, the NAbs levels appear to decline slowly, at an average rate of −0.11 per day. 

Regarding anti-S-RBD antibodies (Figure 2B), the median RC_1–8_ and RC_8–22_ were 0 and 1.87, respectively, indicating the absence of a response to vaccination. However, in the period D22–D36, the median RC_22–36_ value was increased to 151.48 indicating a very high rate of anti-S-RBD production equal to 151.48 units/mL/day. After D36, anti-SRBDs started to fall with a median rate RC_36–50_ equal to −20.3 units/mL/day and from D50 this rate of decline became lower (median RC_50–111_ = −8.1 units/mL/day). This finding is in line with the results from Figure 1B, although anti-S-RBDs declined at a lower rate compared to the NAbs. Multi-group comparison (using the Friedman’s test) and paired comparisons (with the Wilcoxon’s test) led to p-values less than 5% for all RC estimates. 

### 3.5. Correlation between NAbs and Anti-SRBDs

**Figure 3:**
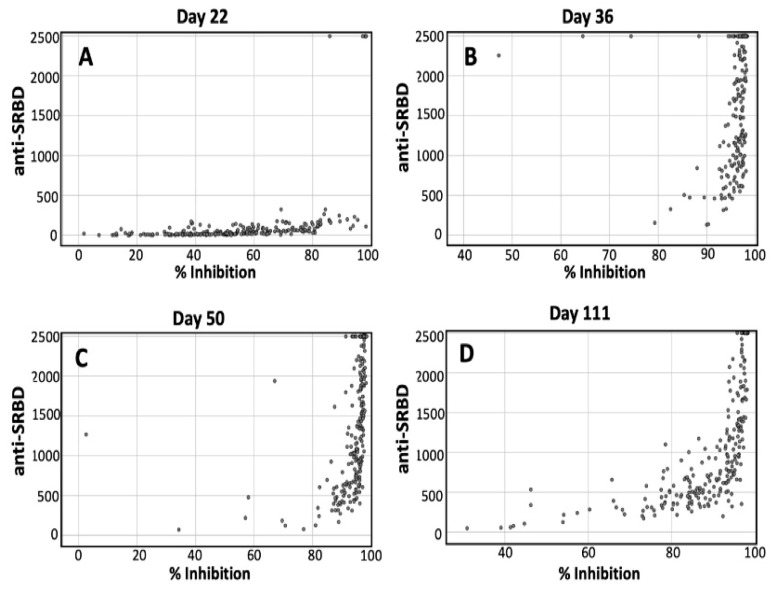
Relationship between the inhibition (%) of SARS-CoV-2 binding to the human host receptor angiotensin-converting enzyme-2 and the Spike-receptor binding domain antibodies (anti-SRBD) at four different occasions (days). (**A**) Day 22, (**B**) Day 36, (**C**) Day 50, (**D**) Day 111.

To elaborate on the relationship between the NAb and anti-SRBD levels, their co-plot was constructed (Figure 3 is a scatter plot between NAbs (horizontal axis) and anti-S-RBD titers (vertical axis)). There was an almost linear relationship between NAbs and anti-S-RBD at D22 (Spearman’s rho correlation coefficient equal to 0.718). However, their relationship becomes non-linear from D36; this is due to the steep increase in anti-S-RBD levels that was observed during the D22–D36 period, while the corresponding increase rate for NAbs was much lower (Figure 1 and Figure 2). Also, the decline of the anti-S-RBD titers was lower compared to that of the NAbs. The composite effect of these functions leads to the non-linear pattern shown in Figure 3B–D. 

### 3.6. Predictive Factors for Antibody Response over Time

The influence of several other factors on NAbs and anti-S-RBD titers has also been studied. Age, medical history (i.e., comorbidities) of each subject, gender, BMI, and medications administered, were examined to determine if they could have an effect on antibody levels on each day or if they could possibly influence the observed drop after D36. With the exception of age and medical history, none of the other factors were found to have a statistically significant effect on NAbs values. In addition to the above, it was investigated whether NAbs titers on D36, D50, and D111 were higher in subjects with positive PCR tests, namely those who already had COVID-19. Of the 283 participants, 17 had a positive PCR test before the first vaccination and the remaining 266 were negative. It is well known that previous infection with SARS-CoV-2 leads to a higher increase in inhibitory levels from D1 to D36, a finding that was also observed in our study. At D8, the mean titers were 16.0 and 39.7 for the PCR-negative and positive subjects, respectively. This shows the strong influence of the previous COVID-19 on the production of NAbs. This effect remains eminent at D22, where the mean inhibitory values are still much higher (65.6 vs. 54.1) for the previously PCR+ subjects. In addition, the possible association between NAbs levels and the occurrence of an adverse event and/or the type of adverse event was investigated. However, no statistically significant association was found between safety problems and NAbs levels, either during the increasing or decreasing period. It is worth noting that such a relationship was not even observed on D1, D8, and D22, which were close to the vaccination time point. In the same context, such a significant relationship, between adverse events and the rate of change was not found in any time interval. All above-mentioned results are presented in Tables S9–S12 of the supplementary material.

A similar analysis of all these factors and their possible impact on anti-S-RBD levels was made. Again, no influence of all these factors was identified with the exception of age and positive PCR test. The role of age is described in the following section, while for those with positive PCR, significantly higher levels are observed at the initial phase (D1, D8) (Mann-Whitney *p*-values < 0.001) and at D111 (*p* = 0.046 < 0.05). No significant differences were observed the other days, but from D50 there is a trend for a slower decline rate for those with PCR+. 

**Figure 4:**
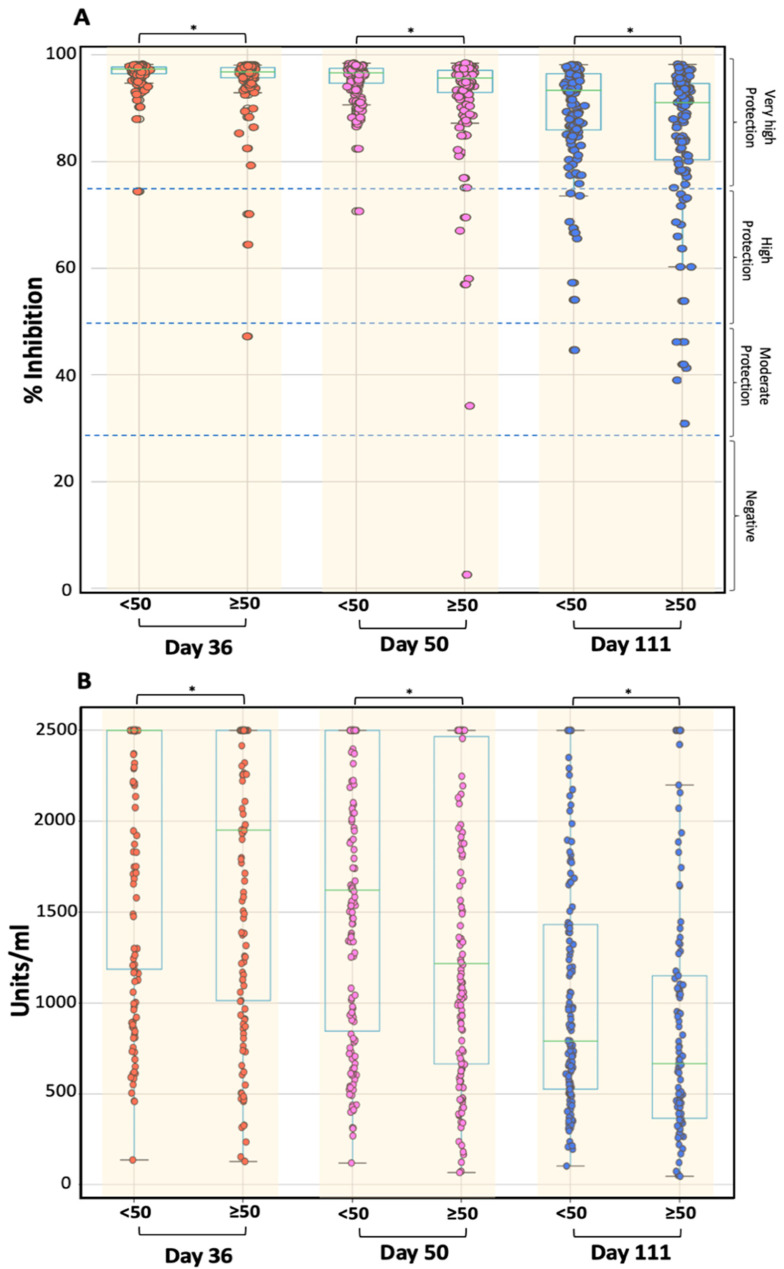
Inhibition (%) of SARS-CoV-2 binding to the human host receptor angiotensin-converting enzyme-2 for the two age groups: <50 years and ≥50 years. Neutralizing antibodies (**A**) and anti-S-RBDs (**B**) levels are shown for days 36, 50, and 111 (3 months after the second shot). Asterisks (*) indicate statistically significant differences (*p*-value < 0.05) between the two compared groups (age < 50 vs. age ≥ 50) at each time point. The boxplot borders refer to the quartiles of the distribution, while the overlaid dots represent the individual values of antibody inhibition. Dashed lines refer to the borderline levels of inhibition i.e., 30%, 50%, and 75%.

Age was found to have a significant effect on NAbs values, according to which young individuals had higher values at D36, D50, and D111. Figure 4A shows the NAbs titers in two age groups on D36, D50, and D111 i.e., during the declining period. Individuals were divided into two classes, those younger than 50 years and those with age greater than or equal to 50 years. Figure 4 clearly shows that younger individuals have higher scores for D36, D50, and D111, which is due to a slower decline in NAbs compared to the older group of subjects (for all three comparisons, Wilcoxon’s *p*-values were <0.05). The same comparison in terms of age group was repeated for the rate of change and the results obtained are in agreement with the above. Indeed, the RC estimates differ significantly between the two age groups (<50 years and ≥50 years) for both the D36–D50 and D50–D111 periods (Wilcoxon *p*-value less than 0.05). It is worth noting that due to the more sensitive nature of RC to detect differences in NAbs values, the influence of age was also statistically significant during the initial rise phase, namely in the intervals D8–D22 and D22–D36 (*p*-values < 0.001 for both intervals). In addition, the same findings, for the role of age in anti-S-RBD levels, were observed (Figure 4B). Younger individuals exhibit significantly higher anti-S-RBD levels at D36, D50, and D111. Using the Mann–Whitney test for comparison of the two groups (<50 and ≥50), the p-values of 0.036, 0.053, and 0.020 were observed for anti-S-RBDs at D36, D50, and D111. Tables S7 and S8, in the Supplementary material, summarize the results for the role of age in the antibodies levels. 

**Figure 5:**
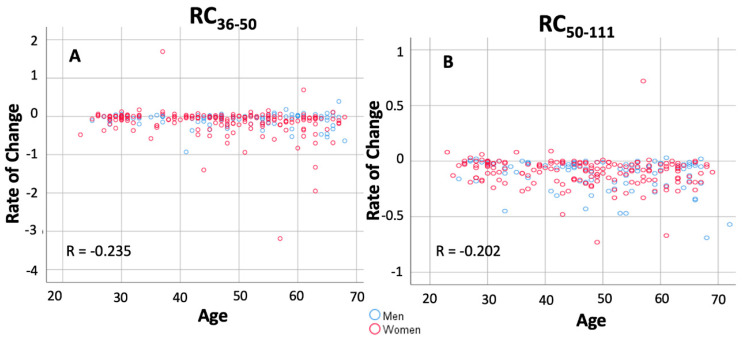
Correlation plots between the rate of change (RC) in the (%) inhibition of SARS-CoV-2 binding and the age of the individuals participated in the study. Age was found to significantly affect RC values for the time intervals 36–50 days (plot (**A**): RC_36–50__,_ *p* = 0.01) and 50–111 days (plot (**B**): RC_50–111__,_ *p* = 0.02). As RC values tend to be higher (in absolute term) values, the change in the % inhibition is greater. Blue circles refer to men, while red circles to women.

To further elucidate the role of age in maintaining high NAbs scores, the correlation between age (treated as a numerical variable) and NAbs scores was examined. Figure 5 shows the results of the correlation analysis between the rate of change from D36 to D111 and age, with the data further stratified by sex. Visual inspection shows that there is a correlation between the rate of NAbs decline and age. In other words, it is clear that older individuals lose NAbs at a higher rate. The correlation coefficients of this relationship are −0.235 and −0.202 for the RC_50–111_ and RC_36–50_, respectively, and the negative sign is due to the decrease in RC scores with increasing age. The larger (in absolute values) correlation coefficient estimates for the D50–D111 period, compared to the D36–D50 period, signify the greater influence of age over time.

**Figure 6:**
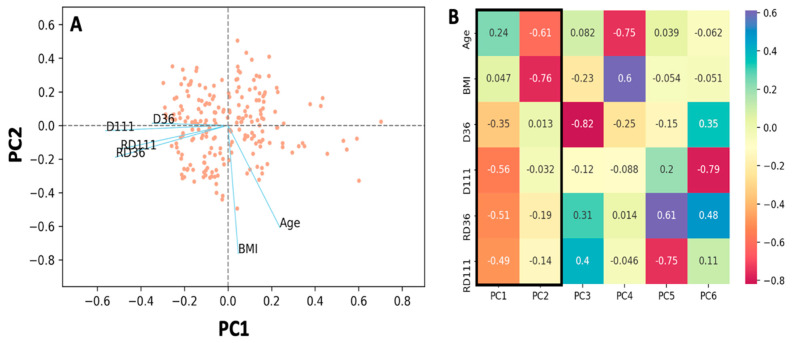
Principal component analysis of the percent inhibition of SARS-CoV-2 binding (D36, D111) and anti-S-RBD levels (RD36, RD111) at two occasions (day 36 and 111) and two subjects’ characteristics: body mass index (BMI) and age. (**A**) Biplot of the two principal components (PC) showing the individual scores and the loadings (blue lines) of the six characteristics. (**B**) Loadings for the entire 6 PCs. The two first PCs, inside the thick border, are plotted in (**A**).

With the aim of investigating the role of age and BMI on NAbs and anti-S-RBD values of the declining phase, principal component analysis was performed. Figure 6A is a PCA biplot for the first two principal components. The first two principal components explain 63.11% of the variability (see Figure S1), while the dots and lines refer to the individual scores and loadings, respectively. The loading scores are also shown in Figure 6B. As the angle between two vectors or between a variable vector and a principal component becomes smaller, their association becomes stronger. In Figure 6A, the NAbs titers on the two different days are close together, indicating their close association. Relatively close to them are also located the anti-S-RBDs. This observation implies that there is some kind of association between the NAbs and anti-S-RBD levels at D50 and D111. Age is in the opposite direction of the horizontal axis, meaning it has a positive loading value with respect to the first principal component. This means that age is negatively associated with the four previously mentioned vectors of antibodies and thus affects them in the opposite way; as age increases, antibody titers become lower. The latter is in line with the results presented above in Figure 4. For BMI, it is clear that it is close to the vertical axis (principal component 2), so the relationship with the horizontal axis is minimal. The latter implies that there is no relationship between BMI and antibody levels, which are close to the horizontal axis. The NAbs and anti-S-RBD values are therefore independent of body mass measures, such as BMI. 

**Figure 7:**
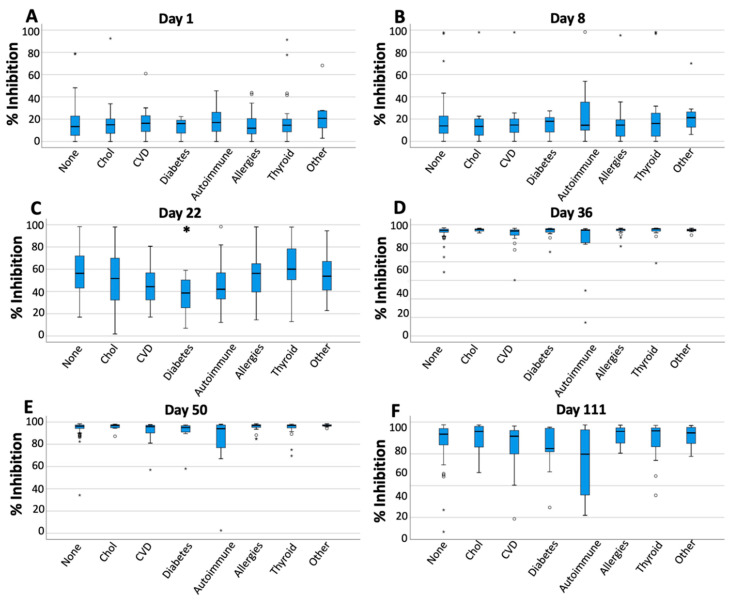
Inhibition (%) of SARS-CoV-2 binding at measurement days (1, 8, 22, 36, 50, 111) divided according to the medical history of the subjects participating in the study. On day 22 (plot (**C**)), subjects with diabetes, cardiovascular problems, and autoimmune diseases had lower inhibition titers compared to the other groups. On this day, the difference was statistically significant (*p* = 0.039) only for subjects with diabetes compared to individuals with no comorbidities. In all other cases, no statistically significant differences were observed. Key: Chol, hypercholesterolemia; CVD, cardiovascular disease. (**A**) Day 1, (**B**) Day 8, (**C**) Day 22, (**D**) Day 36, (**E**) Day 50, (**F**) Day 111; “°” and “*” denote extreme values

Another factor identified as influencing NAbs values was medical history. In this case, the possible influence of comorbidities on the production or elimination of NAbs in individuals was examined. At each time point (i.e., D1, D8, D22, D36, D50, D111), it was investigated whether conditions such as hypercholesterolemia, cardiovascular disease (e.g., hypertension), diabetes, and autoimmune disease could have an impact on NAbs levels. Statistical analysis using the Kruskal–Wallis test revealed a statistically significant difference between comorbidities at D22 (*p*-value = 0.012) and D36 (*p*-value = 0.032). After D36, and especially one and three months after the second vaccination, no significant differences were observed (*p*-values equal to 0.070 and 0.101), but there was a trend where individuals with autoimmune diseases had lower NAbs titers. Figure 7 provides an overall view of the contribution of comorbidities to NAbs inhibition from D1 to D111. On D1, all groups start with almost equal values (Figure 7A). One week after vaccination, the average NAbs values remain similar to D1, but in individuals with autoimmune diseases, the distribution of NAbs values seems to move towards higher values (Figure 7B). At D22, i.e., the day after the second vaccination, subjects with diabetes, cardiovascular problems, and autoimmune diseases have lower NAbs values compared to the other groups (Figure 7C). In subjects with diabetes, this difference was statistically significant (*p* = 0.039) compared to subjects without other comorbidities. No significant differences were found for all other cases (*p* = 0.05). One month after the second vaccination (i.e., D50), no significant differences were found between the groups (Kruskal–Wallis *p*-value = 0.070), although it is clear that the autoimmune group has a greater distribution of NAbs towards lower values (Figure 7D). Finally, three months after the completion of vaccination (D111), the decrease in NAbs values is evident for all groups, but again for the autoimmune group, the dispersion of values is greater and tends towards lower values (Figure 7E). A similar analysis in terms of medical history was also made in the case of anti-SRBD levels. However, no significant results (*p*-values > 0.05) were found. 

### 3.7. Safety Assessment

**Figure 8:**
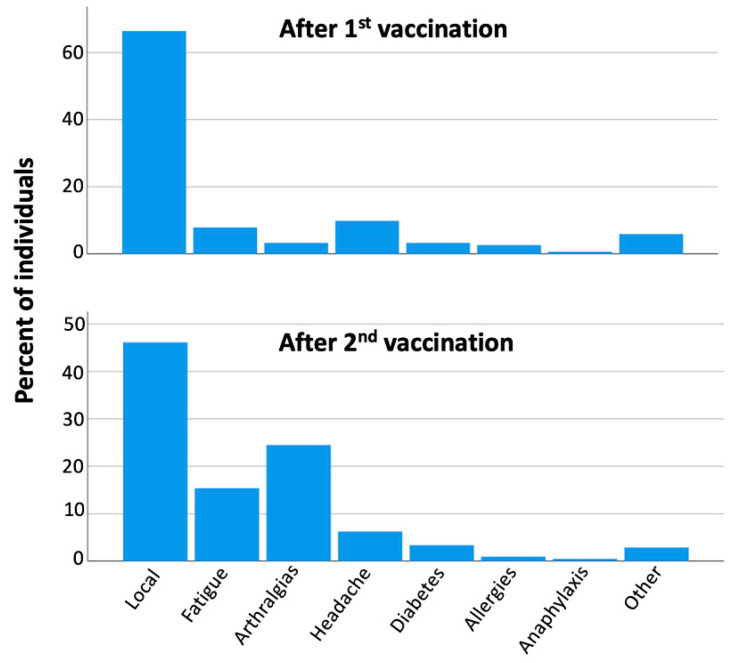
Type of adverse events after the first and second vaccination.

Regarding the safety profile, about half of the subjects (50.67%) had an adverse event after the first vaccination dose. After the second shot, the percentage of subjects with at least one adverse event was 71%. The influence of various factors such as gender, BMI, previous COVID-19, and age on the nature of adverse events was studied after both the first and second vaccination. However, no significant contribution of any factor was found in any of these cases. The type of adverse events, after the first and second vaccination, are shown in Figure 8. The most common adverse event was local manifestations (e.g., pain and/or swelling at the vaccination site, mild limitation of hand movement), followed by fatigue, arthralgias, myalgias, chills, and fever of grade 1 or 2. No grade ≥3 adverse events were observed. No association was found between the type of adverse event and other characteristics of the subjects such as age, sex, etc. 

## 4. Discussion

The aim of this study was to investigate the kinetics of NAbs and anti-S-RBD antibodies against SARS-CoV-2 after over a period of up to three months after the full vaccination with the BNT162b2 mRNA vaccine. It is worth mentioning that for both NAbs and anti-S-RBD antibodies, the maximum levels are observed at D36. A statistically significant decrease in both types of antibodies was observed after D36 up to D111. However, the antibody response persisted above the threshold of positivity at D111. This implies ongoing immune protection against COVID-19 [15,17]. Similarly, a decrease in NAbs levels at day 56 following BNT162b2 has also been recently reported by Favresse et al. [18]. Our results are in line with data regarding the durability of antibody responses both at 3 and 6 months following two-dose vaccination with the mRNA-1273 vaccine [19,20,21]. Although SARS-CoV-2 antibody binding and neutralization responses reduced over time, they remained well above the positivity threshold levels [19,20,21]. The kinetics of antibody responses following vaccination with mRNA-based vaccines are consistent with the kinetics of antibodies against SARS-CoV-2 in convalescent individuals [22,23,24,25]. 

The pattern of antibody kinetics became apparent by the RC index, which addresses the daily change in antibody levels and reflects the rate of antibody production or elimination. Although the first vaccine shot resulted in a positive RC index compared to baseline, a significant increase in the RC index was particularly observed between D22 and D36, which reflected the importance of the second vaccine dose for both NAbs and anti-SRBD production. For NAbs, the RC had an almost constant positive value of 3%/day until D36 but moved to negative values (−0.13/day) after this time point, which may reflect a slow decline in clinical protection against symptomatic COVID-19 at two weeks after the second vaccination [15]. Theoretically, if this rate of decline remained constant, it appears that 5–6 months after the second vaccination, NAbs titers would reach the critical level of 30%, i.e., no longer providing protection, at which time another vaccination might be required. None of the evaluated potential predictive factors, such as BMI, sex, or previous PCR positive test, had a significant effect on NAbs values during the decline period (after D36). In the case of anti-S-RBDs, the RC was almost zero up to D22, but then a steep increase was observed during the D22–D36 period. After D36, anti-S-RBDs started to diminish. Prior history of a PCR+ test led to significantly higher anti-S-RBD titers both at the initial phase (D1, D8) and three months later, as reported previously [21,26,27,28]. 

The age of individuals had a statistically significant influence on both NAbs and anti-S-RBD antibodies. It was found that younger individuals (<50 years) retained higher titers after D36, and their rate of change was significantly lower compared to older subjects (≥50 years). This finding is consistent with the impact of age on the antibody response following vaccination with mRNA-1273 [20].

Furthermore, we found that a previous diagnosis of COVID-19 was associated with enhanced production of NAbs and anti-S-RBD antibodies against SARS-CoV-2. Favresse et al. have evaluated the NAbs response following vaccination with the BNT162b2 at days 14, 21, 28, 42, and 56. In accordance with our results, a stronger response was observed among 30 previously infected compared to 60 COVID-19 naive individuals at all time points [18]. Interestingly, another study by Anichini et al. showed that individuals who have recovered from COVID-19 (*n* = 38) may show a superior humoral response after the first dose of BNT162b2 compared with uninfected individuals who have been fully vaccinated with two doses of BNT162b2 (*n* = 62) [29]. Therefore, a prior COVID-19 diagnosis may be used as a prioritization criterion for the administration of booster vaccine doses.

Regarding the role of gender in the various comparisons in this analysis, it should be noted that unequal sample sizes of men and women were used. The number of women is about twice that of men. In general, unequal sample sizes can lead to biased comparisons, but in our situation, this is not a problem because the imbalance is not high but rather reasonable (67.1% vs. 32.9%) and the Mann–Whitney test can work well with unequal sample sizes. The only limitation is that the statistical power may be reduced, but this does not change the results, and only makes it harder to identify our results.

The presence of comorbidities may impair the anticipated immunogenicity following COVID-19 vaccination [30,31,32,33]. Relatively lower NAbs titers were observed among individuals with diabetes at D22. A larger number of individuals in each subgroup might be necessary in order to reveal additional significant differences in antibody kinetics over time.

In conclusion, we report a persistent but declining anti-SARS-CoV-2 humoral immunity at 3 months following vaccination with BNT162b2 in healthy individuals. Our longitudinal study is ongoing in order to determine the time point of NAbs reduction below the positivity threshold, and the waning of protective immunity against COVID-19 when a booster vaccine dose might be necessary.

## Figures and Tables

**Table 1:** Characteristics of individuals included in the study.

Characteristics	Value
Number of subjects	283
Age (median, range interval) (years)	48 (23–72)
Age < 50 (n, %)	142 (50.2%)
Age ≥ 50 (n, %)	141 (49.8%)
Women (n, %)	190 (67.1%)
Men (n, %)	93 (32.9%)
BMI (median, IQR)	24.8 (5.7)
Underweight (n, %)	11 (3.9%)
Normal weight (n, %)	135 (47.7%)
Overweight (n, %)	95 (33.6%)
Obese (n, %)	42 (14.8%)
PCR+ (n, %)	17 (6.0%)

n, number of subjects; BMI, body mass index; IQR, inter-quartile range; PCR+, positive PCR test for COVID-19.

## Data Availability

The data presented in this study are available on request from the corresponding author.

## Supplementary Materials

The following are available online at https://www.mdpi.com/article/10.3390/cells10081942/s1, Table S1. Normality testing of the Nabs levels on all measurement days; Table S2. Normality testing of the Rate of change (in case of the Nabs levels) on all measurement days; Table S3. Normality testing of the anti-S-RBDs levels on all measurement days; Table S4. Normality testing of the Rate of change (in case of the anti-S-RBDs levels) on all measurement days; Table S5. Wilcoxon signed rank test results for the comparisons of Nabs levels; Table S6. Wilcoxon signed rank test results for the comparisons of rate of change (in case of the Nabs levels); Table S7. Wilcoxon signed rank test results for the comparisons of anti-S-RBDs levels; Table S8. Wilcoxon signed rank test results for the comparisons of rate of change (in case of the anti-S-RBDs levels); Table S9. Association between adverse events and Nabs levels. The Mann-Whitney test results indicate no significant difference between the group of subjects experiencing an adverse event and the group of people without any adverse events; Table S10. Association between adverse events and rate of change in the case of Nabs levels. The results of the Mann-Whitney test show no significant difference between the group of subjects with an adverse event and the group of subjects without an adverse event; Table S11. Association between adverse events and anti-S-RBDs levels. The results of the Mann-Whitney test show no significant difference between the group of subjects with an adverse event and the group of subjects without an adverse event; Table S12. Association between adverse events and rate of change in the case of anti-S-RBDs levels. The results of the Mann-Whitney test show no significant difference between the group of subjects with an adverse event and the group of subjects without an adverse event; Figure S1. Scree plot for the final PCA model showing the proportion of variance explained by each component.
