# Supplementary material for: Kinetics of Anti-SARS-CoV-2 Antibody Responses 3 Months Post Complete Vaccination with BNT162b2; A Prospective Study in 283 Health Workers
Source: Cells. 2021 Jul 30;10(8):1942. doi: 10.3390/cells10081942 (PMC8394923; doi:10.3390/cells10081942)
Supplement: Supplementary file 1 [file cells-10-01942-s001.zip › cells-1305055-supplementary.pdf]

# Supplementary Material: Kinetics of anti-SARS-CoV-2 antibody responses 3 months post complete vaccination with BNT162b2; a prospective study in 283 health workers

**Evangelos Terpos[1#], Ioannis P. Trougakos[2#], Vangelis Karalis[3], Ioannis Ntanasis-Stathopoulos[1], Sentiljana Gumeni[2], Filia Apostolakou[4], Aimilia D. Sklirou[2], Maria Gavriatopoulou[1], Stamatia Skourti[1], Efstathios Kastritis[1], Eleni Korompoki[1], Ioannis Papassotiriou[4] and Meletios A. Dimopoulos[1]**

[#]*Equal contribution as first authors*

## A. Normality testing:

### A1. Nabs levels

**Table S1**. Normality testing of the Nabs levels on all measurement days.

| | Kolmogorov-Smirnov | | Shapiro-Wilk | |
|---|---|---|---|---|
| | **Statistic** | **p-value** | **Statistic** | **p-value** |
| D1 | 0.103 | 0 | 0.888 | 0 |
| D8 | 0.166 | 0 | 0.703 | 0 |
| D22 | 0.047 | 0.02 | 0.988 | 0.034 |
| D36 | 0.325 | 0 | 0.329 | 0 |
| D50 | 0.308 | 0 | 0.381 | 0 |
| D111 | 0.182 | 0 | 0.777 | 0 |

**Table S2**. Normality testing of the Rate of change (in case of the Nabs levels) on all measurement days.

| | Kolmogorov-Smirnov | | Shapiro-Wilk | |
|---|---|---|---|---|
| | **Statistic** | **p-value** | **Statistic** | **Sig.** |
| Rate of change 1_8 | 0.222 | 0 | 0.596 | 0 |
| Rate of change 8_22 | 0.15 | 0.02 | 0.99 | 0.017 |
| Rate of change 22_36 | 0.149 | 0.02 | 0.988 | 0.046 |
| Rate of change 50_111 | 0.182 | 0 | 0.8 | 0 |
| Rate of change 36_111 | 0.18 | 0 | 0.806 | 0 |

## A2. Anti-S-RBDs

**Table S3**. Normality testing of the anti-S-RBDs levels on all measurement days.

| | Kolmogorov-Smirnov | | Shapiro-Wilk | |
|---|---|---|---|---|
| | **Statistic** | **p-value** | **Statistic** | **p-value** |
| R_D1 | 0.508 | 0 | 0.08 | 0 |
| R_D8 | 0.54 | 0 | 0.174 | 0 |
| R_D22 | 0.402 | 0 | 0.275 | 0 |
| R_D36 | 0.277 | 0 | 0.791 | 0 |
| R_D50 | 0.162 | 0 | 0.892 | 0 |
| R_D111 | 0.156 | 0 | 0.884 | 0 |

**Table S4**. Normality testing of the Rate of change (in case of the anti-S-RBDs levels) on all measurement days.

| | Kolmogorov-Smirnov | | Shapiro-Wilk | |
|---|---|---|---|---|
| | **Statistic** | **p-value** | **Statistic** | **p-value** |
| Rate of change 1_8 | 0.54 | 0 | 0.175 | 0 |
| Rate of change 8_22 | 0.374 | 0 | 0.213 | 0 |
| Rate of change 22_36 | 0.222 | 0 | 0.843 | 0 |
| Rate of change 1_36 | 0.247 | 0 | 0.798 | 0 |
| Rate of change 36_50 | 0.141 | 0 | 0.901 | 0 |
| Rate of change 50_111 | 0.095 | 0 | 0.956 | 0 |
| Rate of change 36_111 | 0.11 | 0 | 0.957 | 0 |

## B. Nabs comparison among days 36, 50, 111

**Table S5**. Wilcoxon signed rank test results for the comparisons of Nabs levels.

| | D36 - D111 | D50 - D111 | D36 - D50 |
|---|---|---|---|
| **Z score** | -12.843 | -12.814 | -9.2 |
| **p-value** | < 0.001 | < 0.001 | < 0.001 |

## C. Anti-S-RBDs among days 36, 50, 111

**Table S6**. Wilcoxon signed rank test results for the comparisons of rate of change (in case of the Nabs levels).

| | R_D1 - R_D8 | R_D8 - R_D22 | R_D22 - R_D36 | R_D36 - R_D50 | R_D50 - R_D111 |
|---|---|---|---|---|---|
| **Z score** | -2.38 | -12.976 | -13.034 | -11.441 | -12.032 |
| **p-value** | 0.017 | < 0.001 | < 0.001 | < 0.001 | < 0.001 |

## D. Nabs per age group among days 36, 50, 111

**Table S7**. Wilcoxon signed rank test results for the comparisons of anti-S-RBDs levels.

| | D36 | D50 | D111 |
|---|---|---|---|
| Wilcoxon | 14886 | 16939.5 | 13564 |
| Z score | -2.997 | -3.244 | -3.12 |
| **p-value** | 0.003 | 0.001 | 0.002 |

## E. Anti-S-RBDs per age group among days 36, 50, 111

**Table S8**. Wilcoxon signed rank test results for the comparisons of rate of change (in case of the anti-S-RBDs levels).

| | R_D1 | R_D8 | R_D22 | R_D36 | R_D50 | R_D111 |
|---|---|---|---|---|---|---|
| Wilcoxon | 19377.5 | 17206 | 12447 | 14750 | 16116 | 10542 |
| Z score | -0.45 | -0.289 | -3.074 | -2.092 | -1.936 | -2.331 |
| **p-value** | 0.653 | 0.773 | 0.002 | 0.036 | 0.053 | 0.02 |

## F. Principal Component Analysis

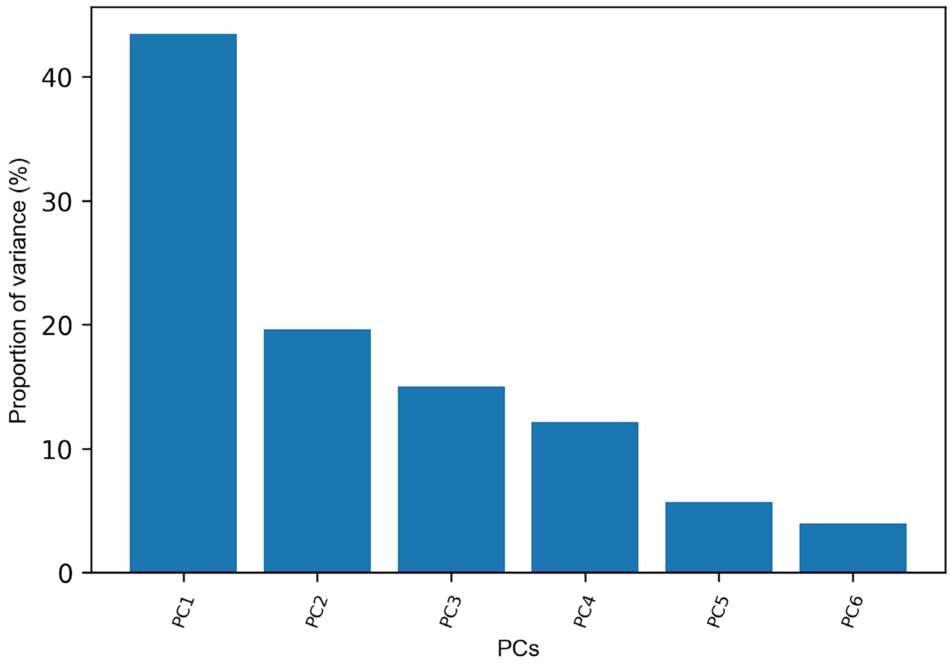


**Figure S1**. Scree plot for the final PCA model showing the proportion of variance explained by each component.

## G. Relationship between adverse events and Nabs (or anti-S-RBDs) levels

### G1. Nabs vs. Adverse events

**Table S9**. Association between adverse events and Nabs levels. The Mann-Whitney test results indicate no significant difference between the group of subjects experiencing an adverse event and the group of people without any adverse events.

| | D1 | D8 | D22 | D36 | D50 | D111 |
|---|---|---|---|---|---|---|
| **Mann-Whitney** | 7710.5 | 7285 | 8507 | 8251.5 | 9708 | 7645.5 |
| **Z score** | -1.837 | -1.233 | -0.46 | -1.282 | -0.439 | -1.426 |
| **p-value** | 0.066 | 0.217 | 0.646 | 0.2 | 0.661 | 0.154 |

### G2. Rate of Change of Nabs vs. Adverse events

**Table S10**. Association between adverse events and rate of change in the case of Nabs levels. The results of the Mann-Whitney test show no significant difference between the group of subjects with an adverse event and the group of subjects without an adverse event.

| | Rate of change1_8 | Rate of change8_22 | Rate of change22_36 | Rate of change1_36 | Rate of change36_5 |
|---|---|---|---|---|---|
| **Mann-Whitney** | 7473 | 6597.5 | 7209 | 7005 | 7362 |
| **Z score** | -0.587 | -1.831 | -0.954 | -1.107 | -1.746 |
| **p-value** | 0.557 | 0.067 | 0.34 | 0.268 | 0.081 |

### G3. Anti-S-RBDs vs. Adverse events

**Table S11**. Association between adverse events and anti-S-RBDs levels. The results of the Mann-Whitney test show no significant difference between the group of subjects with an adverse event and the group of subjects without an adverse event.

| | R_D1 | R_D8 | R_D22 | R_D36 | R_D50 | R_D111 |
|---|---|---|---|---|---|---|
| **Mann-Whitney** | 7878 | 7513 | 7555.5 | 8144 | 8612 | 6123.5 |
| **Z score** | -1.848 | -0.989 | -0.69 | -0.709 | -0.785 | -1.048 |
| **p-value** | 0.065 | 0.322 | 0.49 | 0.479 | 0.433 | 0.295 |

### G4. Rate of Change of Anti-S-RBDs vs. Adverse events

**Table S12**. Association between adverse events and rate of change in the case of anti-S-RBDs levels. The results of the Mann-Whitney test show no significant difference between the group of subjects with an adverse event and the group of subjects without an adverse event.

| | Rate of change 1_8 | Rate of change 8_22 | Rate of change 22_36 | Rate of change 1_36 | Rate of change 36_50 | Rate of change 50_111 | Rate of change 36_111 |
|---|---|---|---|---|---|---|---|
| **Mann-Whitney** | 6888 | 6028 | 5993.5 | 6693 | 7226 | 6032 | 5474 |
| **Z score** | -0.57 | -1.257 | -1.474 | -0.486 | -0.487 | -0.47 | -0.205 |
| **p-value** | 0.568 | 0.209 | 0.14 | 0.627 | 0.626 | 0.639 | 0.837 |
